# Obstructing Duodenal Diffuse Large B-cell Lymphoma with Peritoneal Lymphomatosis with Exceptional Response to R-CHOP

**DOI:** 10.7759/cureus.4621

**Published:** 2019-05-08

**Authors:** Mazen Zaarour, Christopher Busack, Reinhold Munker

**Affiliations:** 1 Hematology and Oncology, Tulane University School of Medicine, New Orleans, USA; 2 Internal Medicine, Tulane University School of Medicine, New Orleans, USA

**Keywords:** small bowel neoplasms, gastrointestinal lymphoma, peritoneal lymphomatosis

## Abstract

Primary small bowel tumors are uncommon and usually carry a poor prognosis. Adenocarcinoma is the predominant histological type while lymphoma is far less common. Small bowel diffuse large B-cell lymphoma (DLBCL) can mimic adenocarcinoma; thus, the distinction can be challenging before tissue examination is performed. Bowel obstruction, as well as peritoneal involvement, are often seen in gastrointestinal (GI) adenocarcinoma cases; however, these features are extremely uncommon with GI lymphomas. Herein, the authors report an unusual case of an obstructing duodenal mass with peritoneal involvement, which was highly suspicious for an advanced duodenal adenocarcinoma. Surprisingly, sampling of the tumor revealed a diffuse large B-cell lymphoma.

## Introduction

Primary small bowel neoplasms are rare tumors with an overall poor prognosis. The predominant histological type is adenocarcinoma. Other less common histologies include carcinoid, lymphoma, and sarcoma [1-2]. These neoplasms often have similar clinical, radiologic, and morphologic features making the distinction difficult without tissue examination.

Despite accounting for up to 40% of cases of extranodal non-Hodgkin's lymphoma (NHL), gastrointestinal (GI) lymphoma is considered an uncommon disease [3]. Diffuse large B-cell lymphoma (DLBCL) is the predominant pathological subtype [4]. The stomach is involved in approximately half of the cases, whereas the small bowel and particularly duodenal involvement is an unusual site of disease.

Clinically, a wide range of nonspecific symptoms can be associated with small bowel lymphoma, with abdominal pain being the most common [5]. While bowel obstruction is a possible manifestation of the disease, a review of the literature favors the jejunum or the ileum as sites of the obstruction rather than the duodenum [6-7]. Furthermore, peritoneal involvement by lymphoma, known as peritoneal lymphomatosis, is less common in small bowel lymphoma [8-9].

In this report, the authors present an unusual case of small bowel obstruction secondary to a duodenal mass with accompanying peritoneal involvement. Surprisingly, the diagnosis was not adenocarcinoma, but DLBCL.

## Case presentation

A 40-year-old Hispanic woman was admitted to University Medical Center New Orleans, Louisiana in August 2017 for evaluation of progressively worsening abdominal pain of eight months duration with associated intermittent nausea and vomiting. The patient also reported losing approximately 30 kilograms in weight. She denied any associated fever, night sweats, change in bowel habits, rash, enlarged lumps or palpable masses. She was previously healthy and denied any personal or family history of malignancy. She never smoked and used alcohol sparingly. She had no known allergies.

On physical exam, the patient’s body temperature was 98.6°F, blood pressure was 100/60 mmHg, and heart rate was 120/min. Cardiovascular and pulmonary exams were unremarkable. The abdomen was soft and mildly tender. A palpable mass was appreciated in the right periumbilical region. No cervical, axillary or inguinal lymphadenopathy was appreciated. Laboratory analysis showed severe metabolic derangements as follows: sodium of 121 mEq/L (reference range, 135-145 mEq/L), potassium of 2.2 mEq/L (reference range, 3.5-5.5 mEq/L), blood urea nitrogen of 61 mg/dL (reference range, 7-20 mg/dL), and serum creatinine of 3.03 mg/dL (reference range, 0.6-1.2 mg/dL). Liver and pancreatic enzymes were in the normal limits. The hematologic panel was consistent with mild normocytic anemia with a hemoglobin of 11.2 g/dL along with leukocytosis with a white blood cell count of 16.6 × 10^9^/L. Platelet count was normal at 299 × 10^9^/L. A peripheral blood smear was within normal limits. A chest radiograph was unremarkable. A non-contrast computed tomography (CT) scan of the abdomen revealed fluid-filled distention of the stomach and proximal duodenum with concern for proximal small bowel obstruction.

**Figure 1:**
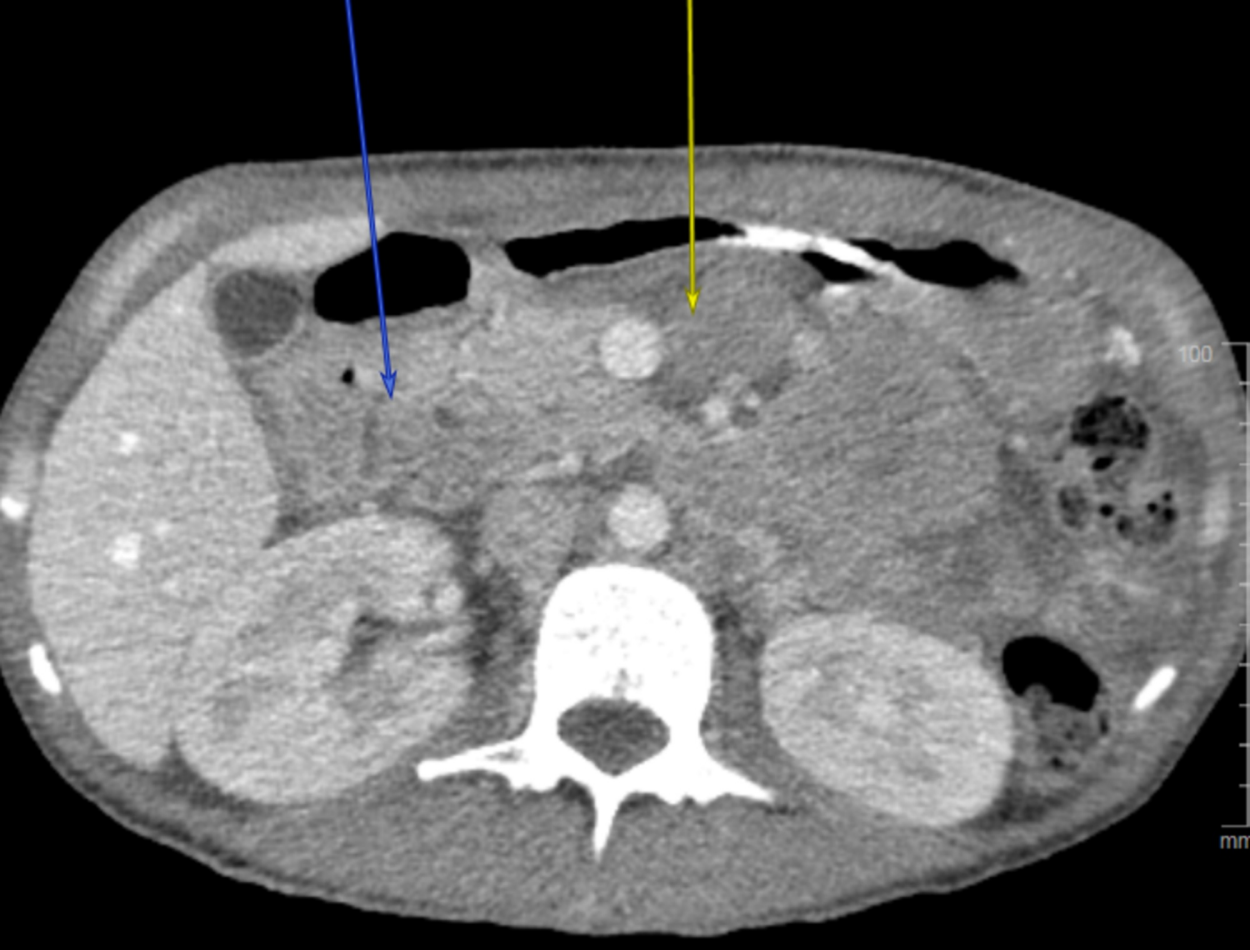
Peritoneal lymphomatosis Computed tomography image showing multifocal masses throughout the mesentery and centered within the peritoneum, compatible with “carcinomatosis.”

The patient was admitted to the intensive care unit given her various metabolic disturbances and was treated conservatively for partial small bowel obstruction. After optimization of her kidney function, a repeat scan of the abdomen with contrast was performed. An irregular mass-like thickening at the level of the second/third portion of the duodenum was identified resulting in severe luminal narrowing. Also, peritoneal "carcinomatosis" with multiple target lesions was noted (Figure 1). The overall picture was concerning for metastatic carcinoma. Later, serum carcinoembryonic antigen (CEA) and Ca 19-9 levels were checked and were within normal limits.

**Figure 2:**
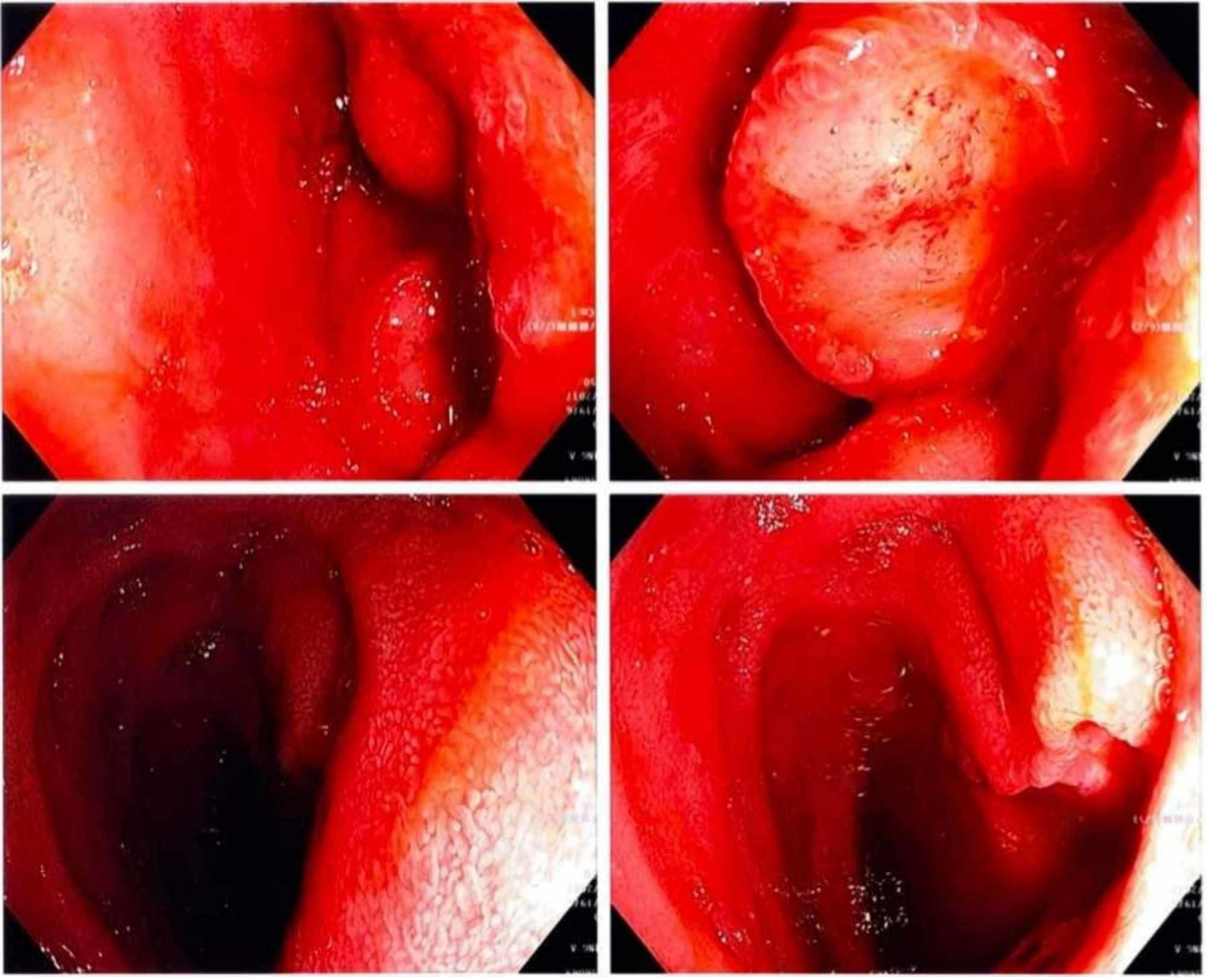
Duodenal mass Frond-like friable obstructing mass in the second portion of the duodenum as seen from different angles on upper endoscopy.

**Figure 3:**
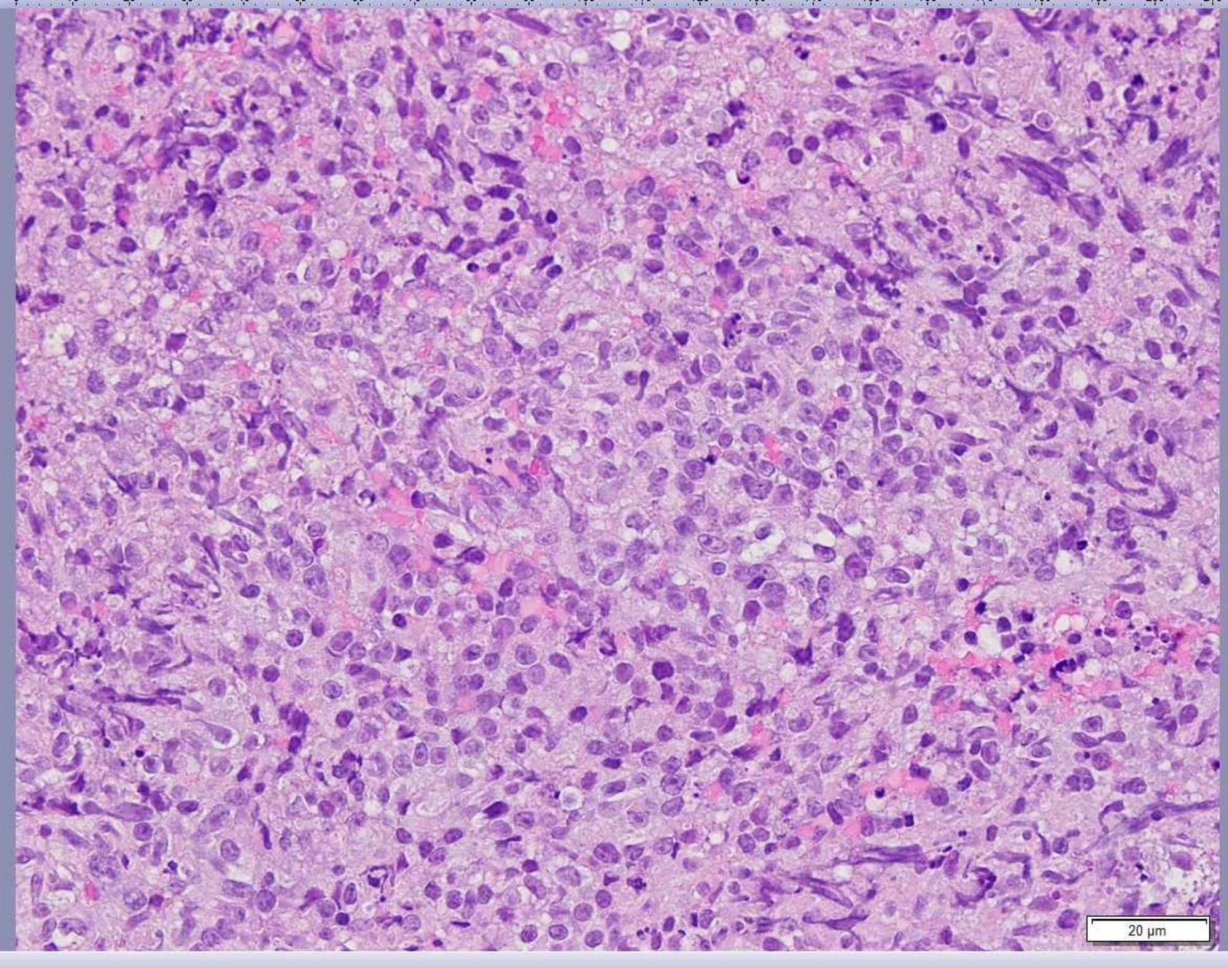
Duodenal mass biopsy Hematoxylin and eosin stain demonstrating sheet of large-sized lymphocytes immunophenotypically consistent with diffuse large B-cell lymphoma.

An upper endoscopy was performed the following day revealing a frond-like friable obstructing mass in the second portion of the duodenum of which biopsies were taken (Figure 2). Large-sized lymphocytes were seen (Figure 3).

Immunohistochemical stains demonstrated that these cells were strongly and diffusely positive for cluster of differentiation (CD)20 and CD79a, and negative for CD3, CD5, CD10, CD30, B-cell lymphoma 2 (Bcl-2) and pan-cytokeratin. Additionally, nuclear staining in about 40% of the neoplastic cells was present for c-Myc and in more than 30% of cells for Bcl-6. A diagnosis of DLBCL with germinal center immunophenotype was made (CD10 negative, Bcl-6 positive, MUM-1 negative). The Ki-67 index was elevated within the B cell population at 60%-70%. No assay specific abnormalities were detected by Bcl-2, Bcl-6, and c-Myc fluorescence in situ hybridization (FISH) probes. Completion staging with neck, chest, and pelvic CT scans failed to demonstrate any additional evidence of disease. A bone marrow examination showed no involvement by lymphoma. Human immunodeficiency virus (HIV) and hepatitis serologies were negative. Serum lactate dehydrogenase was mildly elevated at 318 U/L. Given the patient’s peritoneal lymphomatosis and weight loss, she was diagnosed with stage IV B lymphoma by Ann Arbor staging.

**Figure 4:**
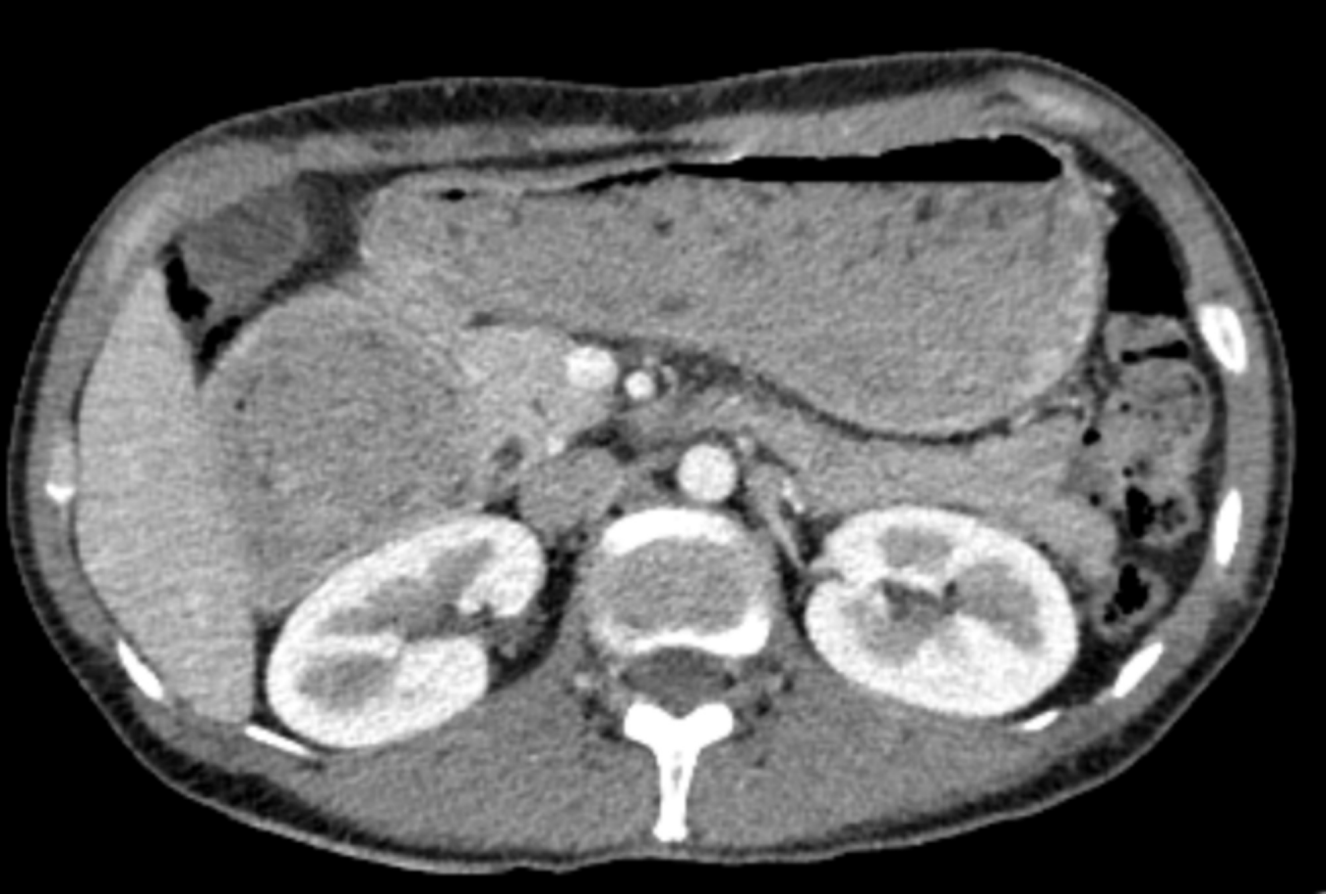
Imaging after completion of treatment Computed tomography image showing resolution of the peritoneal carcinomatosis after 6 cycles of chemotherapy

Given the aggressive presentation with evidence of bowel obstruction and peritoneal involvement (along with markedly elevated Ki-67), the decision was made to start treatment as an inpatient. Therefore, cycle 1 of rituximab, cyclophosphamide, doxorubicin, vincristine, and prednisone (R-CHOP) was administered, and the patient tolerated the treatment well. Central nervous system (CNS) analysis and chemoprophylaxis were not performed as she was considered intermediate-risk for CNS involvement. A few days after the completion of cycle 1, she was able to tolerate oral feeding and she was discharged home. She received the remainder of her treatment as an outpatient and completed a total of six cycles of systemic chemotherapy. She exhibited significant clinical and radiographic improvement throughout her treatment. In fact, a repeat scan after six cycles showed improved luminal duodenal narrowing with resolution of the peritoneal deposits (Figure 4). A push enteroscopy was also performed to assess for local response and demonstrated no evidence of disease. She remains now disease-free 15 months after her diagnosis and 10 months after completion of her treatment.

## Discussion

Primary small bowel neoplasms are rare tumors with an overall poor prognosis. Lymphomas are found in only 20% of small bowel neoplasms and tend to carry a better prognosis than small bowel adenocarcinomas [10]. However, they are more aggressive compared to other GI lymphomas, such as gastric lymphomas [11]. Due to the low incidence rate of this disease, most of the knowledge with respect to small bowel lymphoma derives from small and retrospective studies.

The GI tract is the predominant site of extranodal NHL accounting for up to 40% of cases [12]. Approximately 80%-90% of GI lymphomas are of B-cell origin, with DLBCL being the most common pathological subtype [4]. The pathogenesis of GI lymphomas remains unknown. Nonetheless, a number of risk factors have been implicated such as immunodeficiency syndromes, HIV, inflammatory bowel disease and post-transplant [5,7]. Half of the cases of GI lymphomas involve the stomach, and one-third of cases involve the small bowel [4]. In numerous reports of patients with primary intestinal NHL, the ileum was the most common primary site, followed by the jejunum and the duodenum [4,13]. This distribution parallels that of lymphoid tissue in the different segments of the small bowel. Of special interest, duodenal lymphomas are extremely rare, occurring in < 2% of all GI lymphomas [14-15] and less than 20% of intestinal NHL [4,13].

A wide range of nonspecific clinical symptoms has been reported with intestinal lymphomas. Abdominal pain is the main presenting symptom, and B symptoms are not commonly seen [7]. Bowel obstruction is a common disease manifestation, reported in almost 40% of patients with intestinal NHL in one series [5]. The patient described in this report presented with bowel obstruction secondary to a duodenal tumor. This seems to be an unusual site of obstruction for DLBCL. In fact, not a single case of duodenal obstruction was identified among patients with GI lymphoma and bowel obstruction reported in two cases from Turkey by Karadeli et al. [6] and Avci et al. [7].

Peritoneal involvement can be seen with cases of GI adenocarcinoma, known as peritoneal carcinomatosis. Peritoneal involvement by lymphoma, known as lymphomatosis, is extremely uncommon [8-9]. These two entities are indistinguishable by imaging only. Peritoneal lymphomatosis is thought to be associated with aggressive histological subtypes [16]. Our patient developed bowel obstruction secondary to an intraluminal tumor and was found to have evidence of peritoneal involvement. This presentation was highly concerning for an adenocarcinoma, the most common small bowel tumor which is also well known for peritoneal dissemination. Surprisingly, a lymphoma was diagnosed after tissue examination. GI lymphomas, despite being less common, can closely mimic their adenocarcinoma counterparts and should remain in the differential diagnosis of tumors causing bowel obstruction with peritoneal involvement.

Due to the low incidence of intestinal DLBCL, the optimal treatment remains unknown. Options in the form of surgery alone, surgery followed by systemic chemotherapy, chemotherapy alone, or radiation alone have been reported in the literature [11,13]. Surgery was historically considered the mainstay of treatment. However, since lymphomas are highly chemosensitive, resection is rarely used presently unless in circumstances of palliative intent or for management of complications from the disease like perforation or severe bleeding. The most commonly used chemotherapy regimen is CHOP with rituximab. The patient presented in this report was treated with the conventional R-CHOP regimen for a total of six cycles, which led to complete remission. This response is consistent with other reports of patients with duodenal DLBCL treated with the same regimen [17-18].

## Conclusions

DLBCL presenting as a duodenal mass and resulting in small bowel obstruction is a rare occurrence. Moreover, peritoneal involvement by lymphoma is extremely uncommon and remains a major diagnostic challenge to physicians. Therefore, despite being less common than adenocarcinoma, lymphoma should always remain in the differential diagnosis of GI tumors with or without peritoneal spread.
